# Antenna Array Design Based on Low-Temperature Co-Fired Ceramics

**DOI:** 10.3390/mi15060669

**Published:** 2024-05-21

**Authors:** Lu Teng, Zhongjun Yu, Dali Zhu, Chengxiang Hao, Na Jiang

**Affiliations:** 1 Aerospace Information Research Institute, Chinese Academy of Sciences, Beijing 100094, China; 2 Institute of Information Engineering, Chinese Academy of Sciences, Beijing 100093, China; 3 University of Chinese Academy of Sciences, Beijing 100049, China

**Keywords:** LTCC, antenna array, substrate-integrated waveguide, millimeter wave

## Abstract

With the continuous development of wireless communication technology, the frequency band of 6G communication systems is moving towards higher frequencies such as millimeter waves and terahertz. In such high-frequency situations, wireless transmission requires antenna modules to be provided with characteristics of miniaturization, high integration, and high gain, which presents new challenges to the development of antenna technology. In this article, a 4 × 4 antenna array using multilayered low-temperature co-fired ceramic is proposed, operating in W-band, with a feeding network based on substrate-integrated waveguide, and an antenna element formed through the combination of a substrate-integrated cavity and surface parasitic patches, which guaranteed the array to possess the advantages of high integration and high gain. Combined with the substrate-integrated waveguide to a rectangular waveguide transition structure designed in the early stage, a physical array with a standard metal rectangular waveguide interface was fabricated and tested. The test results show that the gain of the antenna array is higher than 18 dBi from 88 to 98 GHz, with a maximum of 20.4 dBi.

## 1. Introduction

With the advancement of modern wireless communication and radar systems, the research focus has shifted towards antenna systems operating in the millimeter wave and terahertz frequency bands. The W-band, ranging from 75 to 110 GHz with a central frequency of 94 GHz, falls within the atmospheric window with excellent atmospheric penetration capabilities, making it ideal for high-frequency communication systems [1]. The wavelength in this band is approximately 3 mm, and due to its wide spectrum, low atmospheric absorption, and high spatial resolution, the W-band wireless communication transmission technology holds significant research value for applications such as high-resolution passive imaging systems, high-precision radars, high-speed communications, and gigabyte point-to-point data transmissions [2]. The performance and gain of antennas play a crucial role in enabling these functionalities.

Various types of antennas, including parabolic reflector antennas, horn antennas, metal waveguide slot antennas, and microstrip patch antennas, have been extensively investigated in microwave and millimeter-wave wireless systems [3,4,5,6,7,8]. However, traditional reflector antennas, horn antennas, and metal waveguide slot antennas face challenges such as a high cost, complex three-dimensional structures, large volumes, and low integration capabilities, limiting their practical use in cost-sensitive commercial applications [3,4,5]. Although antennas based on microstrip lines, like microstrip patch antennas, offer advantages such as low cost, small size, and easy integration, conventional feed networks based on microstrip lines suffer serious losses at discontinuities and curves in the millimeter wave band, leading to low radiation efficiency and high sidelobe levels [6,7,8]. To address these issues, the use of substrate-integrated waveguide (SIW) has gained popularity due to its low loss characteristics similar to metal waveguides and ease of integration similar to microstrip lines [9,10]. SIW is particularly well-suited for antenna feeding networks in high-frequency millimeter wave bands, and has been widely applied to various antennas in research papers in recent years. For example, circularly polarized and dual-polarized slot antennae can be achieved [11,12], and a multi-band design is also optional [12,13]; Ref. [14] realized a compact SIW slot filtering antenna, and Refs. [15,16,17] explored the design of SIW horn antennas.

**Figure 1:**
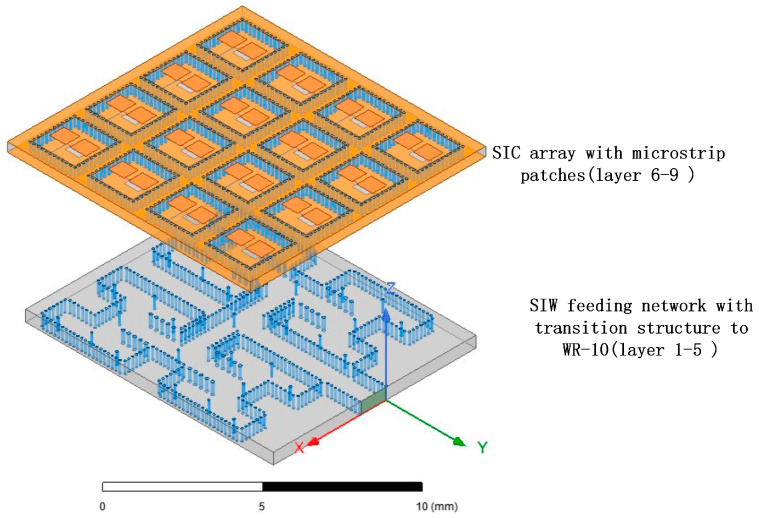
Topology of the antenna array.

Moreover, the substrate-integrated cavity (SIC), derived from SIW, can serve as the radiation unit structure in millimeter-wave antenna arrays. While SIC-based radiation units may exhibit low gain and poor radiation efficiency, the incorporation of high-order mode technology can enhance the antenna array’s performance [18,19,20,21]. In comparison to recent methods involving antenna arrays on Printed Circuit Board (PCB) substrates and ridge waveguide feeding networks fabricated through metal processing [22,23], utilizing low-temperature co-fired ceramics (LTCCs) as the ideal multi-layer ceramic substrate processing technique makes it possible to achieve the integration of SIW-based feed networks and SIC-based antenna unit structures, resulting in a more compact structure. Although 3D-printed or meta-material can provide a convenient design and remarkable radiation performance [24,25], LTCC offers lower costs and increased reliability, and is well-suited for mass production with miniaturization capabilities. Additionally, it eases restrictions on the integration of antenna systems and other processing components. Thus, this paper concentrates on the design of an antenna array utilizing substrate-integrated waveguide (SIW) and substrate-integrated cavity (SIC) technologies, fabricated through the LTCC process to create a W-band antenna array measuring 12 mm × 12 mm × 0.9 mm in size. The topology of the antenna array is depicted in Figure 1.

## 2. Materials and Methods

### 2.1. Design of Substrate-Integrated Waveguide Feeding Network

To achieve high-gain characteristics of an antenna array, a low-loss feeding network is crucial. However, traditional feeding networks based on microstrip lines represent drawbacks such as high loss and discontinuity in the millimeter-wave and terahertz frequency bands. Considering the multi-layer processing capabilities of low-temperature co-fired ceramics, this design selected substrate-integrated waveguide as the foundation to construct the feeding network due to its ease of manufacture and relatively low high-frequency loss.

**Figure 2:**
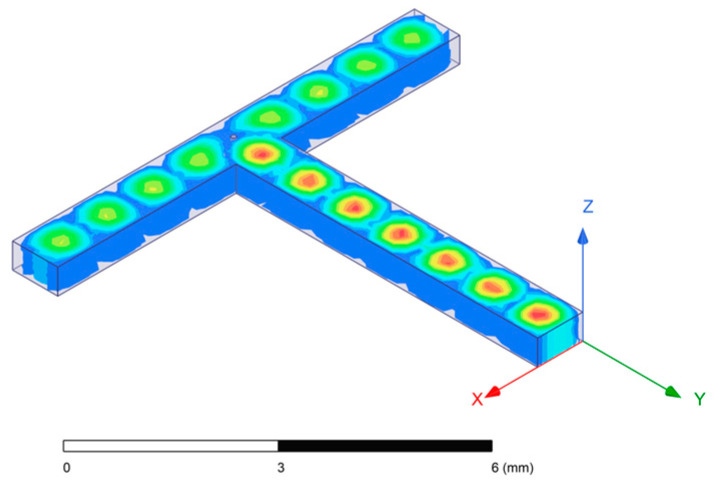
T-junction power divider structure.

The basic structure of the SIW feeding network is a T-junction power divider structure, as shown in Figure 2 (the picture is the ideal model, the actual model needs to replace the side wall with arranged metal vias, and the upper and lower surfaces are coated with metal layers). Additionally, a metal column is included at the T-junction position. By adjusting the position of the metal column, it can effectively regulate the power distribution ratio and the matching characteristics of the two output ports.

Through the multi-stage cascade of T-junctions, power distribution networks of 1–4 and 1–8 can be constructed based on the 1–2 T-junction network. The position of the metal columns and the length of the arms at each stage are adjustable parameters that impact the final power distribution characteristics. The final 4 × 4 array feeding network, as shown in Figure 1, was optimized by utilizing metal vias of various radii to achieve better transmission performance. The different colored cylinders in the figure represent different via sizes.

**Figure 3:**
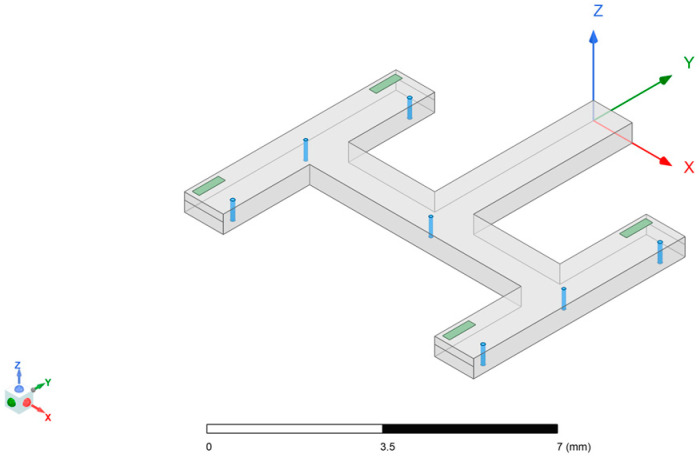
SIW slot feeding structure.

In addition to the SIW power distribution network, consideration must also be given to the feeding structure of the antenna unit. Inspired from the commonly used gap feeding structure in metal waveguides, a slot can be created along the direction of electromagnetic wave transmission at the top of the SIW. Furthermore, a ground metal column can be inserted at the midpoint of the gap on the symmetrical side to enhance the transmission efficiency of the slot, as depicted in Figure 3. This structure, commonly found at the end of SIW feeding networks, has been discussed in existing studies [26,27,28,29].

**Figure 4:**
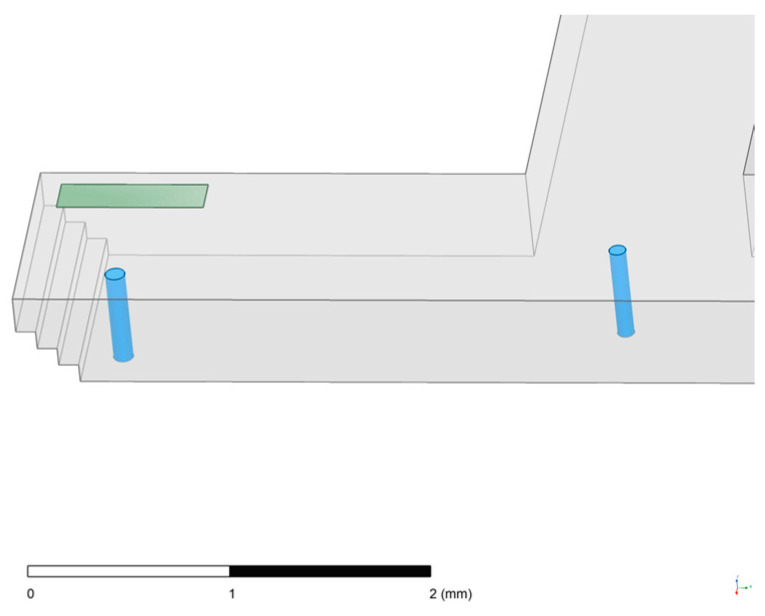
Step structure at the end of the SIW feeding network.

**Figure 5:**
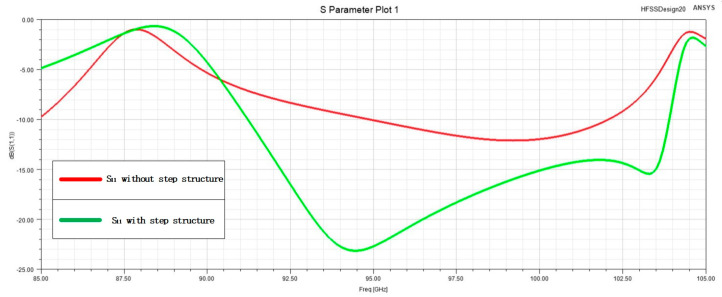
Simulation results of the slot feeding structure.

Considering that this slot feeding structure contains the direction change in the electromagnetic wave transmission, along with the feature of the LTCC multi-layer stacking process, this study introduces a novel stepped structure to enhance the original feeding structure design. This advancement demonstrated improved transmission characteristics for the slit feed, as illustrated in Figure 4 and Figure 5.

### 2.2. Antenna Array Design

#### 2.2.1. Antenna Unit Design

**Figure 6:**
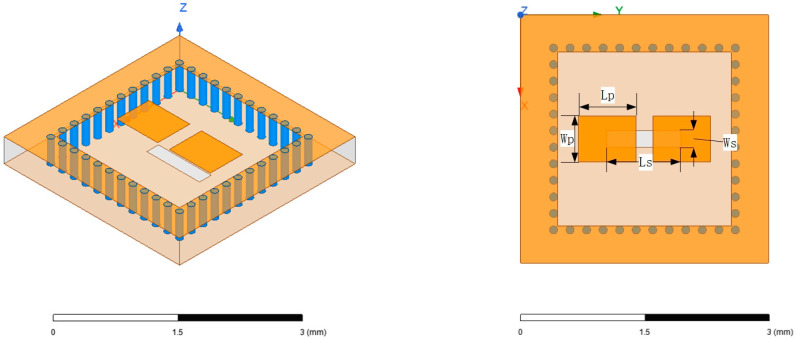
Antenna unit structure.

The antenna unit structure based on substrate-integrated cavity is depicted in Figure 6. It was fabricated using four layers of LTCC substrate, each with a thickness of 0.094 mm. The bottom surface served as a metal layer, with the feeding located at the center slot. Surrounding the slot, there was a ring of metal vias that were strategically arranged.

The basic radiation performance of the SIC antenna unit was influenced by various key parameters, which included the size of the feeding slot, and the shape and dimensions of the cavity. Furthermore, enhancements can be made to the radiation unit, while two primary improvement ideas were proposed. The first suggestion involved adding parasitic microstrip patches atop the cavity to manipulate the radiation mode of the electromagnetic wave, making the radiation power more concentrated to the designed frequency band. The second idea involved excavating a portion of the dielectric material within the cavity to reduce dielectric loss, and in extreme cases, all the dielectric material can be removed to create the substrate-integrated horn antenna. Upon considering the minimum spacing between the excavated cavity boundary and metal via in the LTCC manufacture rules, it was determined through simulation analysis that the second optimization method mentioned above may not have yielded satisfactory results. Nevertheless, during attempts to construct a substrate-integrated horn antenna, expanding the cavity aperture layer by layer was discovered to enhance radiation performance. Consequently, the structure of expanding the aperture layer by layer was adopted in subsequent antenna unit designs.

When incorporating parasitic patches to optimize the antenna unit transmission performance, various parameters such as the number, shape, and positioning of the patches could be adjusted to achieve different optimization effects. The model shown in Figure 6 was the most simplified model with only two patches. The patch size measured Wp = 0.55 mm and Lp = 0.7 mm, with a patch spacing of 0.2 mm, while the length of the feeding slot Ls = 0.9 mm, and the width of the slot Ws = 0.2 mm. In addition, the space between the metal vias was 0.17 mm, which was the minimum value allowed by the LTCC process, and was adopted to achieve optimal performance.

**Figure 7:**
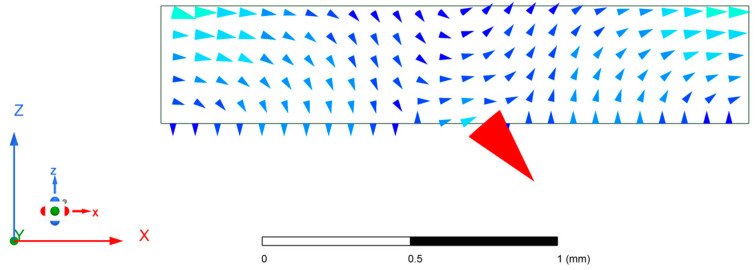
Simulated electric field distribution of the xoz plane in the cavity.

**Figure 8:**
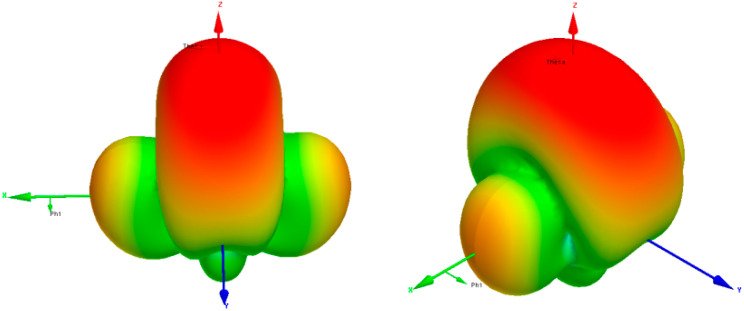
Simulated antenna pattern of the unit.

Under such settings of parasitic patches, the simulated electric field distribution within the cavity is illustrated in Figure 7. This distribution represents the TM_211_ radiation mode, enabling higher radiation gain. The antenna pattern diagram of the antenna unit under this circumstance is shown in Figure 8.

**Figure 9:**
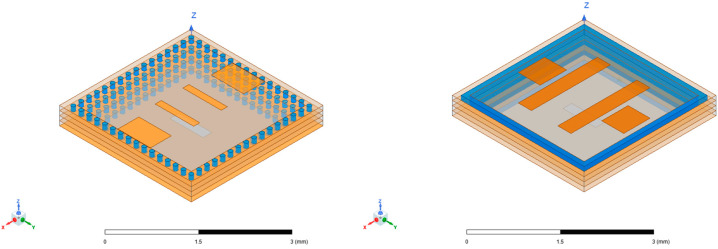
Other patch forms.

The high-order mode patch antenna naturally had a larger electrical size, which was conducive to achieving high gain radiation. However, the high-order mode patch antenna also had defects such as a high sidelobe level and narrow bandwidth [29]. Therefore, this work utilized LTCC substrates with a high dielectric constant (the ceramic green tape material was Ferro A6M, the reference dielectric constant ε_r_ = 6.21 at 95 GHz), alone with symmetric patch design for parasitic radiation cancellation, in order to suppress the sidelobe. Regarding the shape of the parasitic patch, various innovative designs were developed, with two other patch forms shown in Figure 9 for example. These patch forms were based on the same SIC structure, resulting in a relatively similar basic performance. However, due to different intracavity radiation modes resulting from distinct patch designs, there were slight variations in direction pattern and gain stability. These differences will not be extensively compared here, with the subsequent discussion in this paper focusing on the design depicted in Figure 6 as the standard. The reason for this is that the patch form of Figure 6 is the simplest, and with overly complex patch forms it is difficult to ensure the stability of actual testing due to unavoidable LTCC process errors and a limited surface microstrip fabrication accuracy.

#### 2.2.2. Array Simulation and Optimization

**Figure 10:**
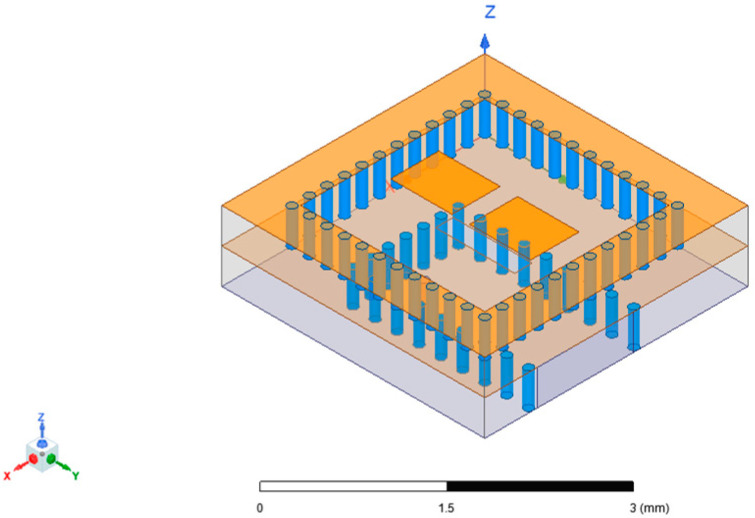
Antenna unit with SIW feeding structure.

Once the antenna unit design have been completed, the next step was to integrate the antenna unit structure with the SIW feeding network for array simulation and optimization. This process involved combining the end structure of the SIW feeding network with the antenna unit. The input port of the simulation model should be adjusted from the slot at the bottom of the SIC to the SIW cross-section, as illustrated in Figure 10. By adjusting the relative position of the slot with the SIW, as well as the length and width of the slot and other parameters, a more optimal transmission effect could be achieved.

On this basis, the 2 × 2 array simulation could be conducted, combining the antenna unit structure with the 1–4 SIW feeding network shown in Figure 3. The main optimization parameters included the spacing between units, the size and position of the feeding slot, etc. Through the previous antenna unit optimization process, a small range of parameter adjustments could be determined. It should be noted that different combinations of translation symmetry and mirror symmetry between antenna elements may result in significant performance differences. Of course, the specific symmetry method can be designed to match the power distribution structure of the feeding network. However, this often requires more vertical space occupation. Therefore, in order to reduce volume and improve integration, the antenna array design in this paper adopted a relatively simplified design in both the feeding network and antenna unit structures, without adding complex matching structures.

**Figure 11:**
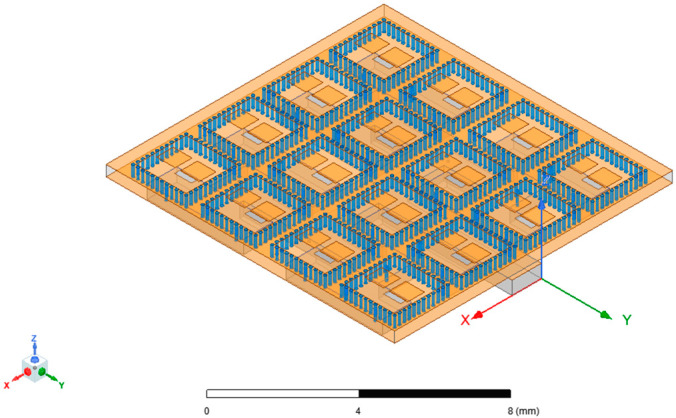
A 4 × 4 array.

After completing the 2 × 2 array simulation, the entire 2 × 2 array can be used as a new unit structure and expanded into a 4 × 4 array through the same method. The intermediate parameter adjustment and optimization process was omitted, and the final 4 × 4 antenna array obtained is shown in Figure 11.

#### 2.2.3. Interface Transition Structure Design

Subsequently, the interface issues in the actual manufacturing and antenna test require further consideration. Prior to this research, the transition structure from substrate-integrated waveguide to Rectangular Waveguide (RWG) was developed [30], in which the SIW-RWG stepped transition structure and the one-to-two transition structure were identified as suitable test interfaces for the antenna array. The design process of these two interface structures is briefly introduced here. As is well known, the WR-10 metallic waveguide is required as testing instrument interfaces in the W-band, which involves the transition structure from the SIW feeding network to rectangular waveguide of WR-10. In the LTCC fabrication process, multiple layers of ceramic green tape are vertically stacked together, then cut and sintered. However, this makes it difficult to guarantee the flatness of the cross-section after sintering. If the feeding interface is designed on the side of the LTCC substrate, the stability of the feeding interface cannot be ensured; therefore, the interface can only be placed on the bottom surface of the antenna array. As a result, a transition structure is needed between the vertically transmitted rectangular waveguide and the horizontally transmitted substrate-integrated waveguide. Specifically, one end of the rectangular waveguide was connected to the bottom surface of the substrate-integrated waveguide, while the SIW bottom metal layer was windowed at the connection point to allow the electromagnetic waves transmitted in the rectangular waveguide to enter the SIW. The size and shape of the window on the contact surface between the SIW and the RWG will affect the transmission characteristics of this type of transition structure. In order to reduce the impact caused by the change in the propagation direction, a stepped structure could be constructed inside the SIW to form a gradual transition, thereby achieving better transmission performance. The thickness of each layer of ceramic green tape in the LTCC process used in this design was fixed, so the overall thickness of the SIW structure could only be an integer multiple of the thickness of a single ceramic layer. Considering the transition of electromagnetic waves from a vertical propagation in the rectangular waveguide to a horizontal propagation in the LTCC substrate, the connection part of the SIW could be equivalent to a section of a dielectric filled waveguide. Therefore, the vertical thickness of the equivalent dielectric filled waveguide should be as close as possible to 1/4λ_g_ in order to achieve better transmission efficiency. After calculation and combining with modeling simulation results, it was appropriate to use five layers of Ferro A6m ceramic green tape with a sintered thickness of 0.094 mm for the SIW transition structure in the W-band, which was the reason for using five layers of LTCC ceramic green tape in the previous SIW feeding network design.

**Figure 12:**
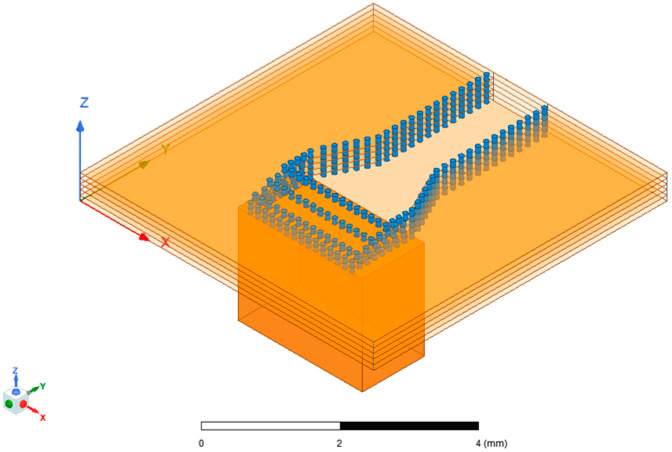
SIW-RWG stepped transition structure.

**Figure 13:**
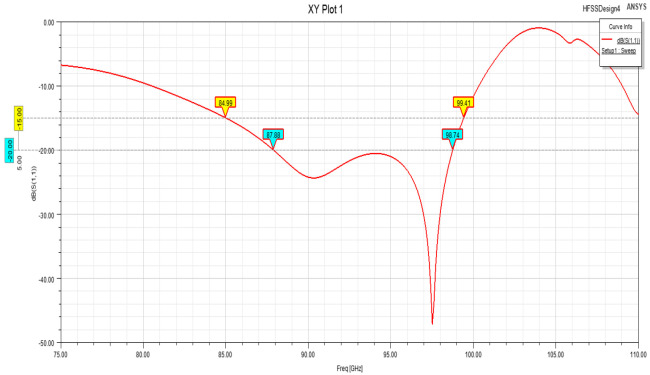
Simulation result of SIW-RWG stepped transition structure.

The designed SIW-RWG stepped transition structure is shown in Figure 12. Considering that the connection part of the substrate-integrated waveguide could be equivalent to dielectric-filled waveguide, and due to the presence of filled dielectric, the dimensions of the dielectric-filled waveguide were smaller than those of a standard rectangular waveguide. This determined the direction of the gradient for the stepped structure, and the highest order of the step should have been less than the number of ceramic layers used. Other design variables, such as the width and length of each step, as well as the size of the window opened on the contact surface between SIW and RWG, needed to be optimized through simulation to determine appropriate values. The final optimized simulation results are shown in Figure 13. The lengths of the four levels of the step structure were 0.02 mm, 0.09 mm, 0.3 mm, and 0.3 mm, respectively, achieving a return loss below −20 dB within a frequency range of 87.8–98.7 GHz. This simulation result exhibited good practicality of the SIW-RWG stepped transition structure in the W-band.

On the basis of the SIW-RWG stepped transition structure, a one-to-two transition structure was further designed to combine the first-stage power distribution structure of the feeding network with the transition structure. The advantage of this one-to-two transition structure is that it can shorten the transmission path and reduce transmission loss. At the same time, since the transition structure was designed on the bottom surface of the substrate-integrated waveguide, it was convenient to add some special structures afforded by the LTCC process to obtain better power distribution and transmission performance. By directly applying symmetrical processing to the SIW-RWG stepped transition structure in Figure 12, a structure similar to a T-junction could be achieved. However, the transmission performance of this primitive structure was not satisfying, and the main problem was the strong reflection of the electromagnetic wave transmission on the contact surface, resulting in a narrow usable bandwidth outside the center frequency point. Referring to the power divider structure of the T-junction in microwave circuits, a special structure could be added on the contact surface to improve the transmission performance. Combined with the special structures supported by the LTCC process, it was decided to set an empty cavity in the SIW substrates connected to the contact surface with the RWG. After simulation analysis and optimization, the shape of the cavity was set to rectangular.

**Figure 14:**
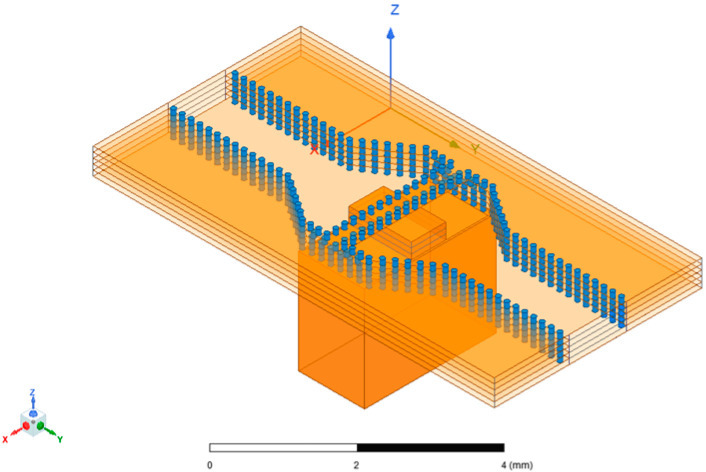
One-to-two transition structure.

The completed design of the one-to-two transition structure is shown in Figure 14. A cavity with a depth of three ceramic layers was excavated on the contact surface between the LTCC substrate and the rectangular waveguide. According to simulation analysis result, the width of the cavity should be set to approximately half of the length in order to achieve better lateral transmission performance. This narrow and elongated shape of the cavity can improve the directionality of electromagnetic wave propagation after entering the LTCC substrate to a certain extent. The specific value of the width can be determined through simulation optimization.

At the same time, a stepped structure was also fabricated on the unexcavated ceramic layers to improve the transmission performance. In addition, the introduction of the air medium in the cavity can bring about resonance frequency changes, which can greatly enhance transmission performance of the one-to-two transition structure. Ideally, the cavity size of each layer of the LTCC substrate should vary gradually, which can achieve a layer-by-layer variation in the equivalent dielectric constant, reducing the reflection to optimize the bandwidth improvement effect of the cavity on the one-to-two transition structure. However, limited by the actual processing difficulty, the cavity size of each layer of the LTCC substrate was consistent in this design.

**Figure 15:**
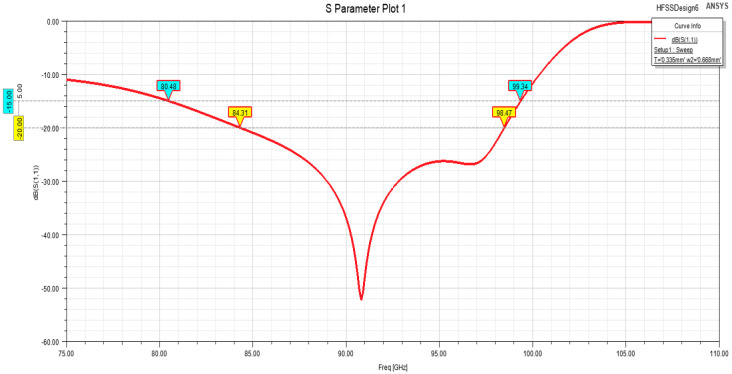
Simulation results of one-to-two transition structure.

The simulation results of the one-to-two transition structure are shown in Figure 15, where the corresponding parameters were set as follows: cavity size of 0.668 mm × 0.335 mm, length of the first step as 0.0425 mm, and length of the second step as 0.1675 mm. It was possible to achieve a return loss of −20 dB or below in the frequency band of 84.3–98.4 GHz. The specific upper and lower frequency limits could be adjusted to achieve slight variations in the frequency band through parameter adjustments.

**Figure 16:**
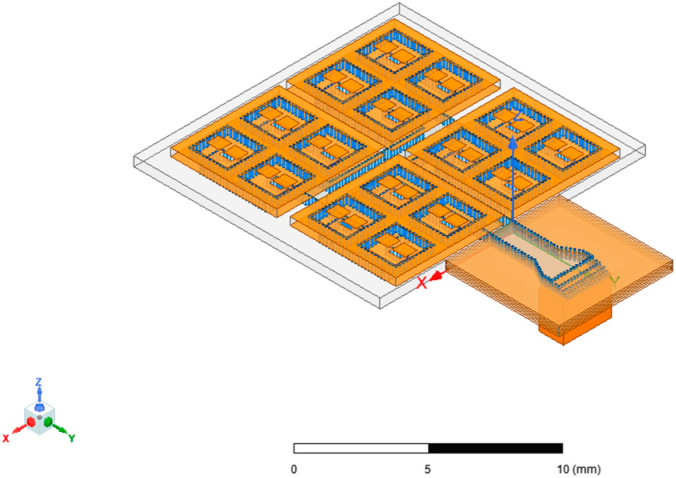
A 4 × 4 array with an SIW-RWG stepped transition structure.

**Figure 17:**
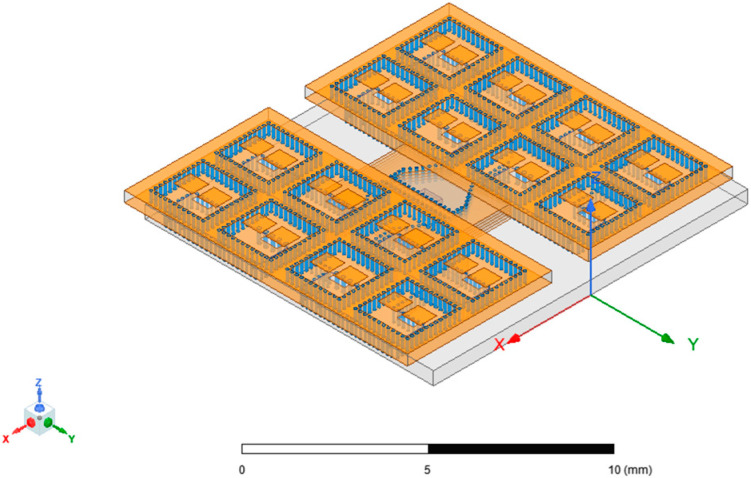
A 4 × 4 array with a one-to-two transition structure.

Figure 16 and Figure 17 show the corresponding models of the SIW-RWG stepped transition structure and the one-to-two transition structure serving as the interfaces for the antenna array.

**Figure 18:**
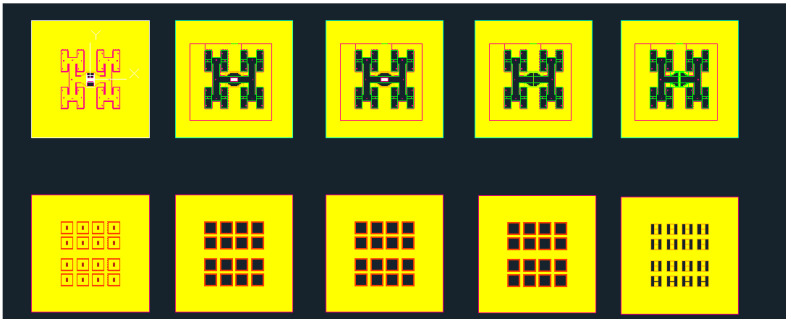
Layout of the LTCC manufacture process.

**Figure 19:**
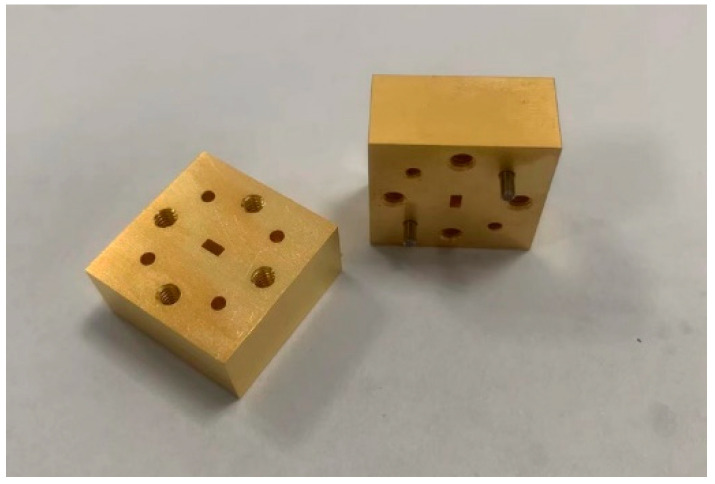
Waveguide adapter.

Upon simulation and comparison, it was determined that the model utilizing the one-to-two transition structure exhibited superior performance. As a result, the subsequent manufacturing and test conducted in this paper were based on this particular model. The layout of the LTCC manufacture process can be seen in Figure 18, while the metal waveguide adapter specifically designed for this study is illustrated in Figure 19. The dimensions of the adapter measured 20 mm × 20 mm × 10 mm, with the central waveguide interface conforming to the standard WR-10, while the flange that pairs with the waveguide port was UG-387.

## 3. Results and Discussion

**Figure 20:**
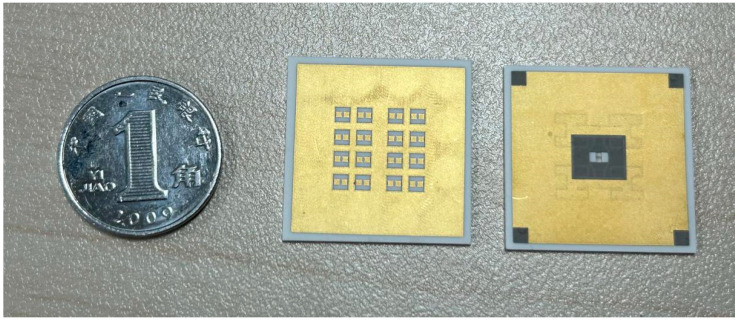
Photograph of the LTCC antenna array.

The finished LTCC antenna array is shown in Figure 20; the external dimensions were 20 mm × 20 mm × 0.9 mm, the actual antenna area was 12 mm × 12 mm, and the surface metal plate area was extended to 20 mm × 20 mm in order to match the external dimension of the waveguide adapter.

Solder paste was utilized for bonding the waveguide adapter to the antenna array. Specifically, a suitable quantity of solder paste was applied to the designated region (indicated in gray) on the rear of the antenna array substrate. The solder paste was then carefully aligned with the waveguide adapter before being placed onto a heated table for bonding under pressure. Subsequently, the solder paste underwent a cooling process to complete the welding procedure.

**Figure 21:**
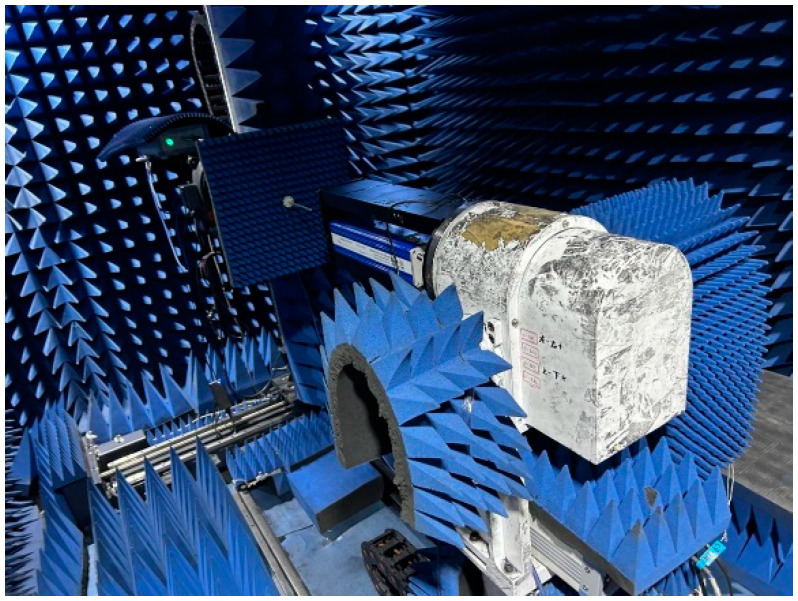
Photograph of the darkroom test conditions.

**Figure 22:**
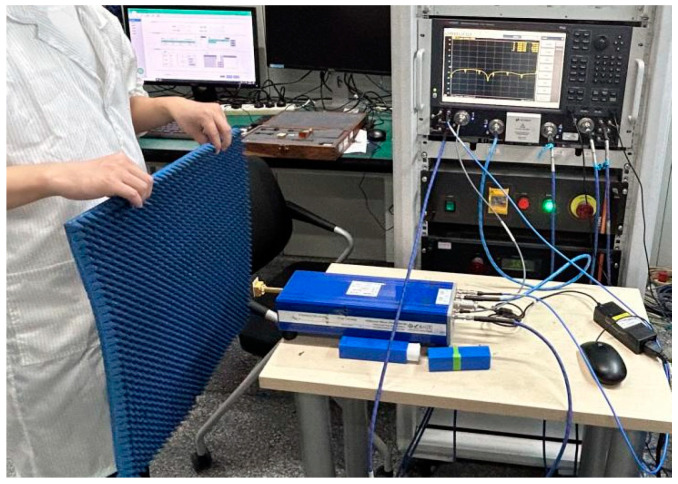
S-parameter test scenario.

**Figure 23:**
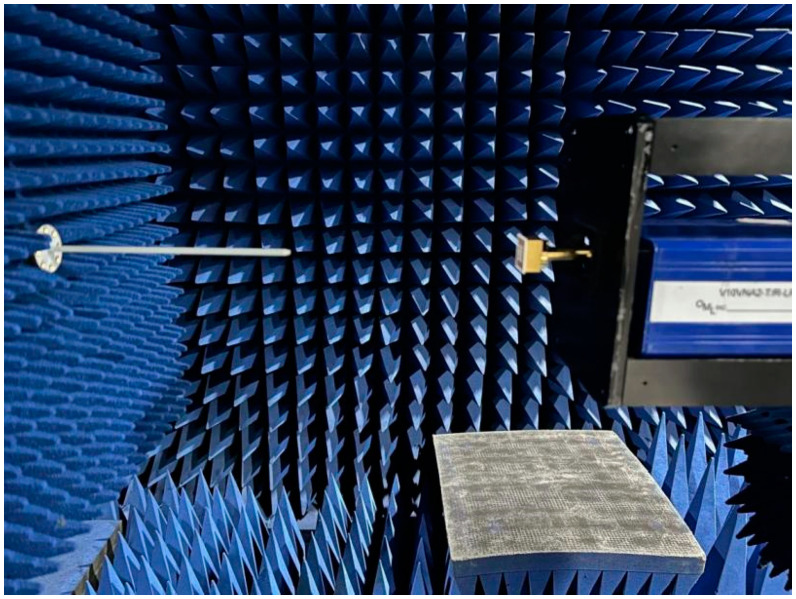
Antenna pattern test scenario.

The darkroom test conditions and scenarios are shown in Figure 21, Figure 22 and Figure 23.

**Figure 24:**
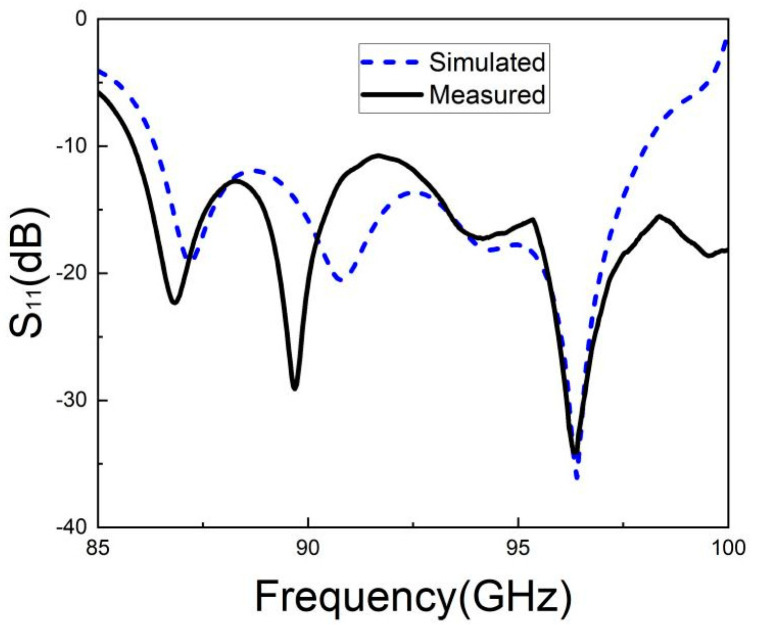
S-parameter test results.

The S-parameter test results of the antenna array are depicted in Figure 24. The measured S_11_ curve was close to the simulated curve, and was less than −10 dB in the target frequency band (88 GHz–98 GHz).

**Figure 25:**
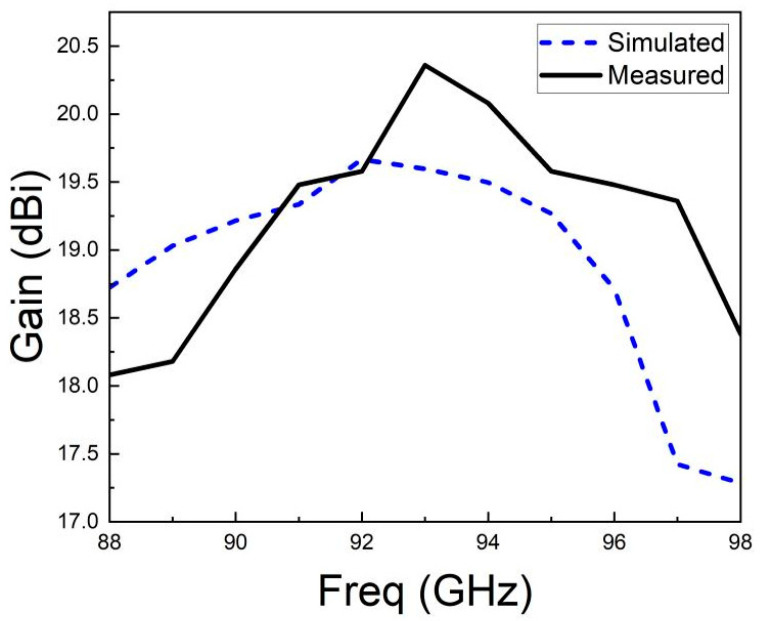
Gain test results.

The gain test results of the LTCC antenna array are depicted in Figure 25. Due to constraints on simulation resources and test conditions, only the peak gain at integer frequency points was simulated and tested. The results indicate that within the target frequency band of 88 GHz−98 GHz, the gain of the antenna array exceeded 18 dBi, reaching as high as 20.4 dBi, with pretty good stability.

**Figure 26:**
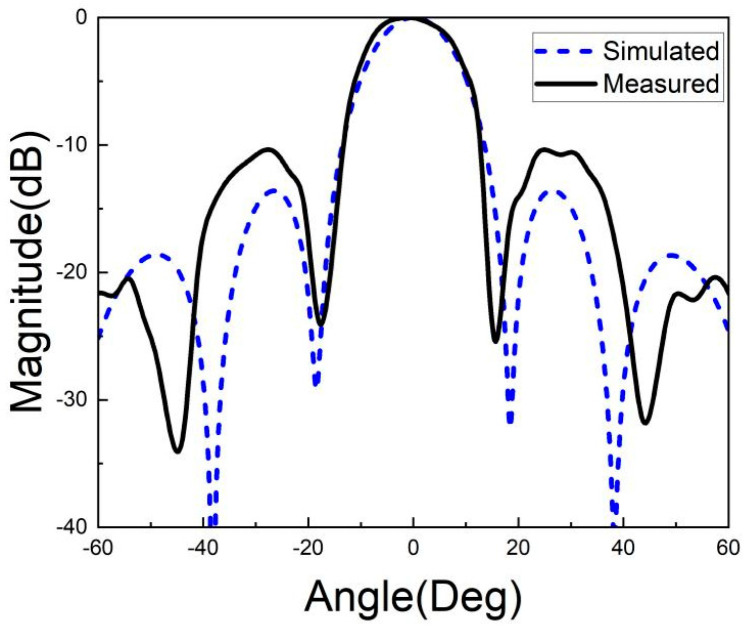
Test results of E-plane radiation pattern at 92 GHz.

**Figure 27:**
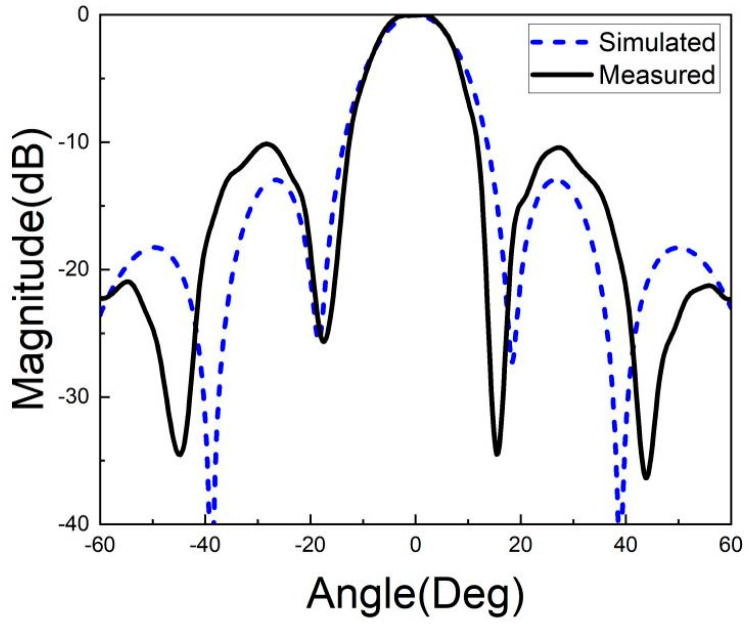
Test results of H-plane radiation pattern at 92 GHz.

**Figure 28:**
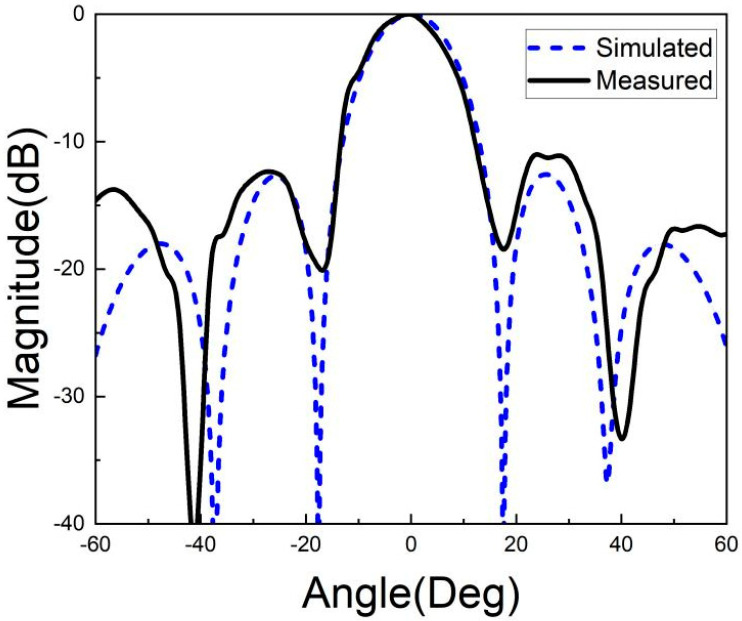
Test results of E-plane radiation pattern at 94 GHz.

**Figure 29:**
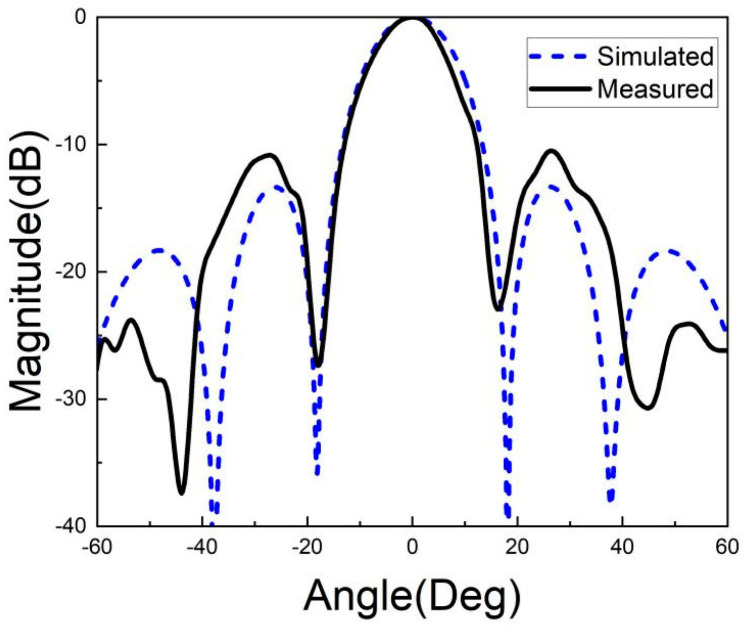
Test results of H-plane radiation pattern at 94 GHz.

**Figure 30:**
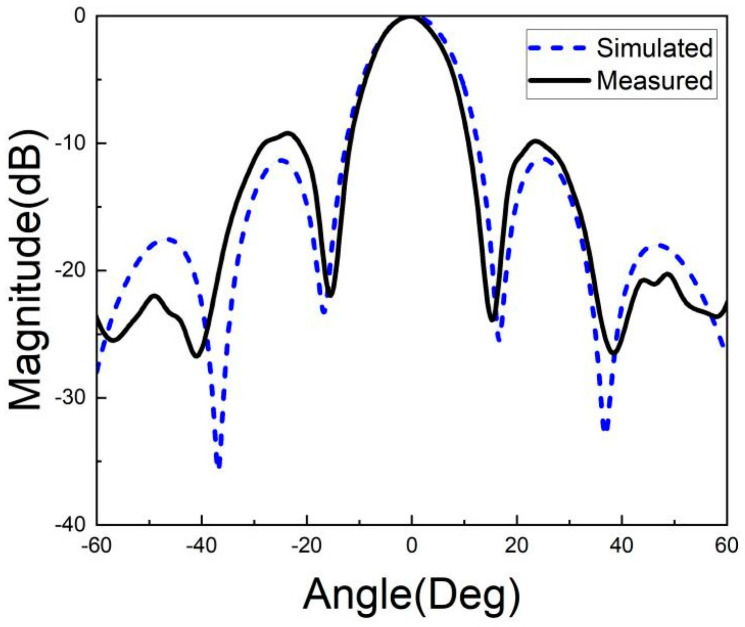
Test results of E-plane radiation pattern at 96 GHz.

**Figure 31:**
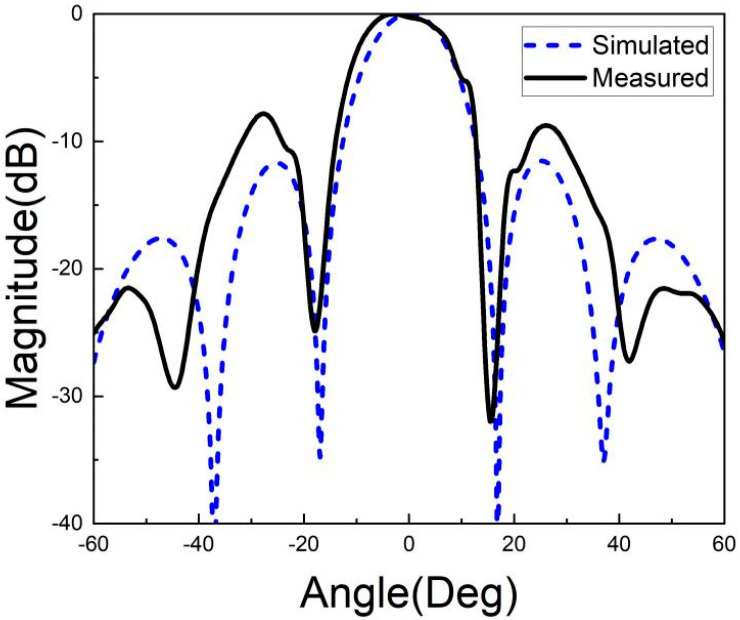
Test results of H-plane radiation pattern at 96 GHz.

Figure 26, Figure 27, Figure 28, Figure 29, Figure 30 and Figure 31 show the test results of the antenna pattern of the LTCC antenna array at 92 GHz, 94 GHz and 96 GHz. Basically, the test results were in good agreement with the simulation results. There exists sidelobe deterioration as expected, but its amplitude is within an acceptable range. 

It is worth noting that the test results were slightly better than the simulation results at certain frequencies. After discussion and analysis, we believe that this can be explained. In simulation settings, material parameters provided in the process file, such as the loss angle tangent, were used, and the worst values were selected to ensure the final performance of the actual antenna array. In addition, in terms of the interface structure, we only conducted physical tests on the D-band before, and there were some defects in the substrate flatness at that time. Therefore, it is highly likely that the performance of the W-band interface structure this time was higher than expected. The most important factor is that in the simulation design of the antenna model, all the side walls composed of SIW were vertically arranged metal columns, and the horizontal metal layer thickness was 0, which is a common setting, because if the metal layer thickness between the dielectric layers were considered, the simulation model would be too complex and consume a lot of simulation resources. In actual LTCC substrates, the thickness of the metal layer between the dielectric layers is about 8 μm, which leads to the actual SIW sidewall being a metal mesh composed of metal columns and thin metal layers, which is closer to the ideal electromagnetic wall and greatly reduces the loss of the entire SIW feeding network. In summary, the difference between the measured results and the simulation results was acceptable.

Table 1 summarizes the performances of several previously published antenna arrays in similar millimeter-wave frequency band, and compares the main parameters with the LTCC antenna array designed in this paper. It can be seen that the LTCC antenna array designed in this paper had the characteristics of a compact structure and high gain.

## 4. Conclusions

In this paper, a W-band high-gain antenna array based on low-temperature co-fired ceramic technology was presented. The feeding network and antenna radiation unit were constructed using a substrate-integrated waveguide and a substrate-integrated cavity. A simplified design approach was implemented to achieve high gain performance, enhance structural compactness, and reduce overall volume. In summary, the presented antenna array exhibited high practicality and a relatively superior performance in the W-band frequency range.

During the antenna design and test, the proposed array was closely integrated with the previously studied and published substrate-integrated waveguide to the rectangular waveguide transition structure. This integration effectively validated the feasibility of the one-to-two transition structure and demonstrated its low-loss characteristics and compact structure, making it particularly suitable for high-gain antenna designs.

In addition, this work demonstrated the practicality of the LTCC technology in the high-frequency band, the impact of processing errors, and that the high-frequency performance degradation of dielectric materials is within an acceptable range.

## Figures and Tables

**Table 1:** Comparison with previously published antenna arrays.

The Literature	Array Element Number	Array Size (mm)	Operating Band (−10 dB)	Maximum Gain (dBi)
[31]	4 × 4	18.6 × 18.6 × 2	57.3–64.0 GHz	18.2
[32]	8 × 8	21.6 × 21.6 × 1.248	87–101 GHz	22.9
[33]	8 × 8	22.8 × 22 × 0.127	94–100 GHz	21.4
[34]	4 × 4	23 × 20 × 0.76	130–152 GHz	16.3
[28]	8 × 8	32 × 20 × 0.818	130.3–145 GHz	20.5
[35]	8 × 16	32 × 18 × 1.413	91.2–96.7 GHz	24.5
This paper	4 × 4	12 × 12 × 0.9	88–98 GHz	20.4

## Data Availability

The datasets utilized and/or examined in this study can be obtained from the corresponding author upon reasonable request.
